# Socio-economic status and overall and cause-specific mortality in Sweden

**DOI:** 10.1186/1471-2458-8-340

**Published:** 2008-09-30

**Authors:** Marianne Weires, Justo Lorenzo Bermejo, Kristina Sundquist, Jan Sundquist, Kari Hemminki

**Affiliations:** 1 Division of Molecular Genetic Epidemiology, German Cancer Research Center (DKFZ), Im Neuenheimer Feld 580, 69120 Heidelberg, Germany; 2 Karolinska Institute, Center for Family and Community Medicine, 141 83 Huddinge, Sweden

## Abstract

**Background:**

Previous studies have reported discrepancies in cause-specific mortality among groups of individuals with different socio-economic status. However, most of the studies were limited by the specificity of the investigated populations and the broad definitions of the causes of death. The aim of the present population-based study was to explore the dependence of disease specific mortalities on the socio-economic status in Sweden, a country with universal health care. Another aim was to investigate possible gender differences.

**Methods:**

Using the 2006 update of the Swedish Family-Cancer Database, we identified over 2 million individuals with socio-economic data recorded in the 1960 national census. The association between mortality and socio-economic status was investigated by Cox's proportional hazards models taking into account the age, time period and residential area in both men and women, and additionally parity and age at first birth in women.

**Results:**

We observed significant associations between socio-economic status and mortality due to cardiovascular diseases, respiratory diseases, to cancer and to endocrine, nutritional and metabolic diseases. The influence of socio-economic status on female breast cancer was markedly specific: women with a higher socio-economic status showed increased mortality due to breast cancer.

**Conclusion:**

Even in Sweden, a country where health care is universally provided, higher socio-economic status is associated with decreased overall and cause-specific mortalities. Comparison of mortality among female and male socio-economic groups may provide valuable insights into the underlying causes of socio-economic inequalities in length of life.

## Background

Socio-economic inequalities in overall and cause-specific mortality have been previously reported for several populations [1-11]. Low socio-economic status has been generally associated with a higher mortality due to cardiovascular and respiratory disease, diabetes and several types of cancer, independently of the socio-economic indicator used (for example, occupation, educational level, income or a combination of these factors) [1,3,12]. By contrast, an excess of breast cancer mortality among women with a high socio-economic status has been noticed in different countries [1-3,13-16].

The direction and magnitude of the difference in length of life and mortality depends on two different components: the time to diagnosis of the disease (age of onset) and the time from diagnosis to death (survival time). Several factors associated with socio-economic disparities in survival have been identified, including treatment discrepancies among socio-economic groups and lower screening compliance in deprived persons, thus leading to socio-economic differentials in the stage of disease and the subsequent prognosis [17-19]. However, data on socio-economic status and mortality are sparse and most studies are limited by the specificity of the investigated populations and by the broad definitions of the causes of death [3,4,6-8,10,16,20]. This study investigates socio-economic differences among the most common causes of death taking advantage of the nationwide Swedish Family-Cancer Database. Although there is some evidence that a socio-economic gradient in cancer survival is present in Sweden, it is still unclear to what extent the socio-economic status influences other disease specific mortalities in a country with universal health care [21,22,3,8,11]. It is important to notice here that the structure of the Database (Swedes born after 1931 with their biological parents), together with the restriction of the analyses to individuals aged 30–60 years in the Swedish census of 1960, resulted in a study population where all individuals were parents.

## Methods

The present study was based on the 2006 update of the Swedish Family-Cancer Database [23]. Statistics Sweden created this family database in 1995 by linking information from national censuses, the Multigeneration Register and the Swedish Causes of Death Register, to the Swedish Cancer Registry using an individually unique national registration number. The present study included women and men who were residing in Sweden in 1960, i.e. present at the 1960 national census. In order to adequately describe the socio-economic status, the individuals' age at the beginning of the follow-up period ranged from 30 to 60 years (birth year between 1900 and 1930). Information on socio-economic status was available for 95% of the individuals. In the Swedish census of population of 1960, socio-economic status was categorized into nine levels [24,25]: blue-collar worker, service worker, farmhand (employee in agriculture), farmer (employer in agriculture), white-collar worker, employer, entrepreneur (company owners in industrial, trade, transport and service sectors), professional (physicians, solicitors etc.) and military personnel.

Information on the main underlying and up to ten contributing causes of death was derived from the Swedish Causes of Death Register. The main underlying causes of death were classified in the four major groups: cardiovascular, respiratory, endocrine-nutritional-metabolic diseases and cancer. Underlying causes of death were coded according to the International Classification of Disease (ICD) revision seven to ten, depending on the year of death [26-29]. In contrast with cause-specific mortality, overall mortality included any cause of death. Mortality due to cardiovascular disease included all deaths due to any cardiovascular disease and was further subdivided into ischaemic heart, cerebrovascular and other forms of heart diseases. Death due to cancer included all deaths due to any cancer and was further subdivided into the following types of cancer: lung, colorectal, stomach, pancreatic, breast and prostate cancers. Respiratory system specific mortality included all deaths due to any respiratory disease and was further subdivided into chronic obstructive pulmonary disease (COPD), influenza and pneumonia. Diabetes was considered a separate group within endocrine, nutritional and metabolic diseases.

The association between overall and disease specific mortality and socio-economic status was investigated by comparing mortality rates/hazard rates between socio-economic groups. These results were summarized by mortality rate ratios/hazard ratios (HRs) using a Cox regression model with age as the underlying time scale (for simplicity later in the text also referred to as mortality) [30]. Analyses were implemented using the PHREG procedure of SAS Version 9.1. Follow-up started for each individual in January 1, 1961. Censoring events were defined as emigration, end of the study (December 31, 2003) or death of any other cause than the investigated disease. Regression models were separately fitted for each cause of death and each sex, and they included the covariates residential area (big cities, south or north of Sweden), time period (by five year categories from 1961 to 2004) and, for women, fertility history (parity and age at first birth). Female and male blue-collar workers constituted the largest socio-economic group and were therefore used as reference category. Spearman correlation coefficients were used to determine the degree of similarity between findings of female and male socio-economic groups separately for each disease.

Spearman correlation coefficients were used as a measure of the within-group similarity in HRs related to different diseases. An estimated correlation value close to one would indicate homogeneous HRs for the investigated causes of deaths within a determined socio-economic group. Relationships between the socio-economic groups were visualized in a hierarchical clustering dendrogram of their average profiles (HCLUST function in R Version 2.5.1[31], average linkage method with 1 – Spearman correlation as distance).

Appropriate human subjects approval and consent forms for the group to construct the database have been secured from the Ethics Committee of the Huddinge University Hospital, Karolinska Institutet. Permit number: Dnr 12/00, March 27, 2000.

## Results

The number of women and men at risk and the number of fatalities by socio-economic status are shown in Table 1. Overall, more than one million women and more than one million men were followed-up; 579,288 women and 760,964 men died during the follow-up period. Female and male overall and cause-specific mortality rate ratios/HRs according to socio-economic status are presented in Table 1 to Table 6.

**Table 1:** Eligible population and overall mortality in the Swedish population from 1960 to 2004: Number of women and men at risk of mortality, number of fatalities, age at death distribution and HRs for overall mortality according to socio-economic status in the Swedish population from 1960 to 2004^1^.

Overall mortality	Women				Men			
		
	Population	Fatalities	Age at death^3^	HR^4 ^(95% CI)	Population	Fatalities	Age at death^3^	HR^5 ^(95% CI)
**All combined^2^**	1025856	579288	78 (56;92)	NA	1060370	760964	75 (54;89)	NA
Blue-collar worker	356190	208011	78 (55;91)	Ref.	465577	341027	74 (54;89)	Ref.
Service worker	73132	48862	79 (57;92)	1.02 (1.00;1.03)	12892	10214	74 (54;84)	**1.04 **(1.02;1.06)
Farmhand	35122	22297	78 (56;92)	**1.07 **(1.05;1.08)	42407	31741	76 (56;90)	**0.94 **(0.93;0.95)
Farmer	108518	70149	80 (58;93)	**0.93 **(0.93;0.94)	109169	84544	77 (58;91)	**0.85 **(0.85;0.86)
White-collar worker	323311	156288	78 (54;92)	**0.83 **(0.83;0.84)	283398	182977	74 (54;89)	**0.87 **(0.86;0.87)
Employer	22585	11880	80 (55;93)	**0.78 **(0.76;0.79)	27644	19532	75 (55;90)	**0.85 **(0.83;0.86)
Entrepreneur	90628	53430	79 (56;92)	**0.90 **(0.89;0.91)	97719	76068	75 (55;89)	0.99 (0.98;1.01)
Professional	10121	5668	80 (55;93)	**0.83 **(0.80;0.85)	11869	8849	75 (54;90)	**0.93 **(0.91;0.95)
Military personnel	6249	2703	77 (53;91)	**0.83 **(0.80;0.86)	9695	6012	74 (52;88)	**0.83 **(0.81;0.85)

^1 ^Bold type, 95% CI does not include 1.00

^2 ^Any cause of death

^3 ^Median age at death with 5% and 95% quantiles

^4 ^Adjusted for residential area, time period and fertility history

^5 ^Adjusted for residential area and time period

HR Hazard Ratio, CI Confidence Interval, NA not applicable

**Table 2:** Cardiovascular disease mortality: Number of fatalities and HRs for cardiovascular disease according to socio-economic status for women and men in Sweden from 1960 to 2004^1^

Cardiovascular disease	Women		Men	
		
	Fatalities	HR^3 ^(95% CI)	Fatalities	HR^4 ^(95% CI)
**Overall cardiovascular disease^2^**				
Blue-collar worker	102389	Ref.	178383	Ref.
Service worker	24416	1.01 (0.99;1.02)	5088	0.98 (0.96;1.01)
Farmhand	11641	**1.10 **(1.08;1.12)	17428	**0.96 **(0.94;0.97)
Farmer	37276	**0.95 **(0.93;0.96)	47133	**0.88 **(0.87;0.89)
White-collar worker	69740	**0.78 **(0.77;0.78)	92567	**0.86 **(0.85;0.87)
Employer	5208	**0.68 **(0.66;0.70)	9860	**0.83 **(0.81;0.84)
Entrepreneur	26247	**0.88 **(0.87;0.89)	40052	0.99 (0.98;1.01)
Professional	2544	**0.74 **(0.71;0.77)	4378	**0.88 **(0.86;0.91)
Military personnel	1093	**0.73 **(0.69;0.78)	2873	**0.78 **(0.76;0.81)
**Ischaemic heart disease**				
Blue-collar worker	50393	Ref.	116367	Ref.
Service worker	12172	**1.03 **(1.01;1.05)	3232	**0.96 **(0.93;0.99)
Farmhand	5808	**1.10 **(1.07;1.13)	11213	**0.94 **(0.92;0.96)
Farmer	17915	**0.92 **(0.91;0.94)	29381	**0.85 **(0.84;0.86)
White-collar worker	32657	**0.75 **(0.74;0.76)	59120	**0.85 **(0.84;0.86)
Employer	2310	**0.63 **(0.60;0.66)	6155	**0.81 **(0.79;0.83)
Entrepreneur	12777	**0.88 **(0.86;0.90)	25884	0.99 (0.98;1.01)
Professional	1192	**0.72 **(0.68;0.76)	2745	**0.86 **(0.83;0.90)
Military personnel	507	**0.69 **(0.64;0.76)	1878	**0.80 **(0.76;0.84)
**Cerebrovascular disease**				
Blue-collar worker	26634	Ref.	29697	Ref.
Service worker	6134	0.98 (0.95;1.01)	850	0.99 (0.92;1.05)
Farmhand	3083	**1.10 **(1.06;1.14)	3031	0.98 (0.94;1.02)
Farmer	10084	0.98 (0.96;1.01)	8610	**0.94 **(0.92;0.96)
White-collar worker	18556	**0.80 **(0.78;0.81)	15861	**0.89 **(0.87;0.90)
Employer	1409	**0.72 **(0.68;0.76)	1759	**0.87 **(0.83;0.91)
Entrepreneur	6952	**0.90 **(0.87;0.92)	6868	1.02 (0.99;1.05)
Professional	656	**0.74 **(0.68;0.80)	775	**0.93 **(0.86;1.00)
Military personnel	301	**0.77 **(0.69;0.87)	408	**0.67 **(0.61;0.74)
**Other forms of heart diseases**				
Blue-collar worker	12925	Ref.	15155	Ref.
Service worker	3050	0.98 (0.94;1.02)	496	**1.11 **(1.02;1.22)
Farmhand	1475	**1.14 **(1.08;1.20)	1637	**1.08 **(1.02;1.13)
Farmer	5153	1.02 (0.99;1.05)	4882	1.02 (0.99;1.06)
White-collar worker	9355	**0.80 **(0.78;0.83)	7903	**0.83 **(0.81;0.86)
Employer	775	**0.75 **(0.70;0.81)	902	**0.81 **(0.76;0.87)
Entrepreneur	3380	**0.88 **(0.84;0.91)	3501	1.00 (0.96;1.04)
Professional	373	**0.81 **(0.73;0.89)	406	**0.91 **(0.82;0.99)
Military personnel	138	**0.74 **(0.63;0.87)	271	**0.82 **(0.73;0.92)

^1 ^Bold type, 95% CI does not include 1.00

^2 ^ICD codes: ICD-7: 400–468, ICD-8: 390–458, ICD-9: 390–459, ICD-10: I00-I99

^3 ^Adjusted for residential area, time period and fertility history

^4 ^Adjusted for residential area and time period

HR Hazard Ratio, CI Confidence Interval, NA not applicable

**Table 3:** Cancer mortality: Number of fatalities and HRs for cancer according to socio-economic status for women and men in Sweden from 1960 to 2004^1^

Cancer	Women		Men	
		
	Fatalities	HR^3 ^(95% CI)	Fatalities	HR^4 ^(95% CI)
**Overall cancer^2^**				
Blue-collar worker	54394	Ref.	85328	Ref.
Service worker	12204	1.03 (0.99;1.04)	2633	**1.09 **(1.05;1.13)
Farmhand	5176	0.99 (0.96;1.02)	7126	**0.88 **(0.86;0.91)
Farmer	16570	**0.94 **(0.92;0.96)	19145	**0.82 **(0.81;0.84)
White-collar worker	47175	**0.93 **(0.92;0.94)	50442	**0.93 **(0.92;0.94)
Employer	3432	**0.90 **(0.87;0.94)	5345	**0.93 **(0.91;0.96)
Entrepreneur	14096	**0.96 **(0.94;0.97)	18937	1.02 (0.99;1.04)
Professional	1572	**0.94 **(0.89;0.99)	2347	1.00 (0.96;1.04)
Military personnel	890	0.94 (0.87;1.01)	1807	0.96 (0.91;1.01)
				
**Lung cancer**				
Blue-collar worker	4410	Ref.	16263	Ref.
Service worker	1152	**1.25 **(1.17;1.33)	495	1.06 (0.97;1.16)
Farmhand	329	**0.84 **(0.75;0.94)	948	**0.66 **(0.62;0.70)
Farmer	773	**0.63 **(0.58;0.68)	1773	**0.43 **(0.41;0.45)
White-collar worker	4201	0.93 (0.89;1.00)	8500	**0.80 **(0.78;0.82)
Employer	297	0.92 (0.82;1.04)	825	**0.79 **(0.77;0.81)
Entrepreneur	1104	0.96 (0.90;1.03)	3315	**0.95 **(0.92;0.99)
Professional	136	0.98 (0.83;1.17)	406	**0.89 **(0.81;0.98)
Military personnel	89	1.01 (0.82;1.25)	355	0.96 (0.87;1.07)
				
**Stomach Cancer**				
Blue-collar worker	2944	Ref.	7014	Ref.
Service worker	719	1.08 (0.99;1.18)	212	1.03 (0.90;1.19)
Farmhand	330	1.10 (0.98;1.23)	711	1.00 (0.93;1.08)
Farmer	1071	1.03 (0.96;1.11)	1942	0.96 (0.91;1.01)
White-collar worker	2092	**0.80 **(0.76;0.85)	3082	**0.74 **(0.71;0.78)
Employer	145	**0.72 **(0.61;0.85)	265	**0.58 **(0.52;0.66)
Entrepreneur	753	0.93 (0.86;1.00)	1444	**0.92 **(0.87;0.98)
Professional	77	0.85 (0.67;1.06)	107	**0.55 **(0.46;0.67)
Military personnel	28	**0.61 **(0.42;0.88)	119	0.86 (0.71;1.03)

^1 ^Bold type, 95% CI does not include 1.00

^2 ^ICD codes: ICD-7: 140–239, ICD-8: 140–239, ICD-9: 140–239, ICD-10: C00-D48

^3 ^Adjusted for residential area, time period and fertility history

^4 ^Adjusted for residential area and time period

HR Hazard Ratio, CI Confidence Interval, NA not applicable

**Table 4:** Cancer mortality (continued): Number of fatalities and HRs for cancer according to socio-economic status for women and men in Sweden from 1960 to 2004^1^

Cancer	Women		Men	
		
	Fatalities	HR^2 ^(95% CI)	Fatalities	HR^3 ^(95% CI)
**Colorectal Cancer**				
Blue-collar worker	6527	Ref.	9186	Ref.
Service worker	1367	0.96 (0.91;1.02)	297	1.14 (1.02;1.28)
Farmhand	620	0.98 (0.91;1.07)	854	0.98 (0.92;1.05)
Farmer	2080	**0.94 **(0.90;0.99)	2201	**0.86 **(0.82;0.90)
White-collar worker	5705	0.95 (0.91;1.00)	5789	1.00 (0.97;1.04)
Employer	434	0.95 (0.86;1.05)	635	1.04 (0.96;1.13)
Entrepreneur	1736	0.97 (0.92;1.02)	2155	**1.07 **(1.02;1.12)
Professional	188	0.92 (0.80;1.07)	284	1.13 (0.99;1.27)
Military personnel	119	1.06 (0.88;1.27)	185	0.92 (0.79;1.06)
				
**Pancreatic Cancer**				
Blue-collar worker	3922	Ref.	5179	Ref.
Service worker	918	1.08 (0.99;1.16)	166	1.13 (0.97;1.32)
Farmhand	368	0.97 (0.87;1.08)	466	0.94 (0.85;1.03)
Farmer	1250	0.96 (0.91;1.03)	1184	**0.86 **(0.80;0.91)
White-collar worker	3501	0.96 (0.92;1.02)	3260	1.00 (0.96;1.05)
Employer	279	1.00 (0.88;1.13)	357	1.07 (0.96;1.19)
Entrepreneur	1010	0.94 (0.88;1.01)	1255	**1.12 **(1.05;1.19)
Professional	103	0.85 (0.70;1.03)	142	1.01 (0.85;1.20)
Military personnel	60	0.88 (0.68;1.14)	111	0.98 (0.81;1.19)
				
**Breast Cancer**				NA
Blue-collar worker	7686	Ref.		
Service worker	1578	0.96 (0.91;1.01)		
Farmhand	660	**0.91 **(0.84;0.98)		
Farmer	2450	1.00 (0.96;1.05)		
White-collar worker	7708	**1.05 **(1.01;1.08)		
Employer	579	1.09 (1.00;1.18)		
Entrepreneur	2086	1.01 (0.96;1.06)		
Professional	264	1.12 (0.99;1.27)		
Military personnel	127	0.98 (0.82;1.14)		
				
**Prostate Cancer**		NA		
Blue-collar worker			14844	Ref.
Service worker			473	**1.12 **(1.02;1.23)
Farmhand			1456	1.00 (0.94;1.05)
Farmer			4783	**1.10 **(1.06;1.14)
White-collar worker			9578	1.02 (1.00;1.05)
Employer			1091	1.05 (0.99;1.14)
Entrepreneur			3415	1.03 (0.99;1.07)
Professional			465	1.12 (1.00;1.23)
Military personnel			338	1.02 (0.92;1.14)

^1 ^Bold type, 95% CI does not include 1.00

^2 ^Adjusted for residential area, time period and fertility history

^3 ^Adjusted for residential area and time period

HR Hazard Ratio, CI Confidence Interval, NA not applicable

**Table 5:** Respiratory disease mortality: Number of fatalities and HRs for respiratory disease according to socio-economic status for women and men in Sweden from 1960 to 2004^1^

Respiratory disease	Women		Men	
		
	Fatalities	HR^3 ^(95% CI)	Fatalities	HR^4 ^(95% CI)
**Overall respiratory disease^2^**				
Blue-collar worker	12654	Ref.	23233	Ref.
Service worker	3211	**1.06 **(1.02;1.10)	759	**1.09 **(1.01;1.17)
Farmhand	1361	**1.09 **(1.03;1.16)	2031	**0.88 **(0.84;0.92)
Farmer	4057	**0.88 **(0.85;0.92)	5449	**0.76 **(0.74;0.78)
White-collar worker	9259	**0.79 **(0.79;0.81)	10901	**0.75 **(0.73;0.77)
Employer	797	**0.80 **(0.75;0.86)	1168	**0.67 **(0.64;0.72)
Entrepreneur	3216	**0.88 **(0.84;0.91)	4727	**0.88 **(0.86;0.91)
Professional	398	**0.90 **(0.81;0.99)	573	**0.82 **(0.76;0.89)
Military personnel	214	1.10 (0.96;1.26)	352	**0.72 **(0.64;0.80)
				
**COPD**				
Blue-collar worker	3559	Ref.	8552	Ref.
Service worker	944	**1.21 **(1.12;1.30)	303	**1.22 **(1.09;1.37)
Farmhand	299	0.93 (0.83;1.05)	688	**0.87 **(0.80;0.94)
Farmer	539	**0.51 **(0.47;0.56)	1418	**0.58 **(0.55;0.62)
White-collar worker	2888	**0.80 **(0.76;0.84)	3967	**0.71 **(0.68;0.73)
Employer	251	0.90 (0.79;1.03)	387	**0.61 **(0.55;0.68)
Entrepreneur	831	**0.86 **(0.80;0.93)	1613	**0.85 **(0.80;0.89)
Professional	115	0.95 (0.79;1.15)	194	**0.78 **(0.67;0.89)
Military personnel	82	1.17 (0.94;1.46)	126	**0.64 **(0.54;0.76)
				
**Influenza and Pneumonia**				
Blue-collar worker	6800	Ref.	10845	Ref.
Service worker	1716	1.01 (0.96;1.07)	348	1.03 (0.93;1.15)
Farmhand	832	**1.17 **(1.09;1.26)	1048	**0.92 **(0.87;0.99)
Farmer	2793	1.00 (0.96;1.05)	3191	**0.87 **(0.84;0.91)
White-collar worker	4728	**0.79 **(0.76;0.82)	5317	**0.81 **(0.79;0.84)
Employer	417	**0.78 **(0.70;0.86)	620	**0.75 **(0.69;0.81)
Entrepreneur	1801	**0.88 **(0.83;0.93)	2373	**0.93 **(0.88;0.97)
Professional	222	0.90 (0.79;1.03)	298	**0.89 **(0.79;0.99)
Military personnel	93	1.05 (0.86;1.29)	158	**0.75 **(0.64;0.88)

^1 ^Bold type, 95% CI does not include 1.00

^2 ^ICD codes: ICD-7: 470–527, ICD-8: 460–519, ICD-9: 460–519, ICD-10: J00-J99

^3 ^Adjusted for residential area, time period and fertility history

^4 ^Adjusted for residential area and time period

HR Hazard Ratio, CI Confidence Interval, NA not applicable

**Table 6:** Endocrine, nutritional and metabolic disease mortality: Number of fatalities and HRs for endocrine, nutritional and metabolic disease according to socio-economic status for women and men in Sweden from 1960 to 2004^1^

Endocrine nutritional and metabolic diseases	Women		Men	
		
	Fatalities	HR^3 ^(95% CI)	Fatalities	HR^4 ^(95% CI)
**Overall endocrine nutritional and metabolic diseases^2^**				
Blue-collar worker	5473	Ref.	5458	Ref.
Service worker	1159	0.94 (0.88;1.00)	195	**1.28 **(1.11;1.48)
Farmhand	697	**1.21 **(1.12;1.31)	582	1.06 (0.97;1.15)
Farmer	1797	**0.86 **(0.81;0.91)	1440	**0.91 **(0.87;0.98)
White-collar worker	2674	**0.56 **(0.54;0.59)	2754	**0.81 **(0.77;0.85)
Employer	163	**0.42 **(0.36;0.49)	288	**0.81 **(0.72;0.91)
Entrepreneur	1153	**0.74 **(0.69;0.79)	1347	**1.12 **(1.06;1.19)
Professional	91	**0.51 **(0.42;0.63)	132	0.91 (0.77;1.08)
Military personnel	45	**0.54 **(0.40;0.72)	67	**0.56 **(0.44;0.72)
				
**Diabetes Mellitus**				
Blue-collar worker	4651	Ref.	4463	Ref.
Service worker	976	0.93 (0.87;1.00)	167	**1.35 **(1.15;1.57)
Farmhand	586	**1.20 **(1.10;1.30)	486	1.09 (0.99;1.19)
Farmer	1525	**0.85 **(0.80;0.90)	1191	**0.93 **(0.87;0.99)
White-collar worker	2056	**0.51 **(0.49;0.54)	2211	**0.79 **(0.75;0.83)
Employer	109	**0.33 **(0.27;0.40)	232	**0.80 **(0.70;0.91)
Entrepreneur	935	**0.70 **(0.65;0.75)	1133	**1.16 **(1.08;1.24)
Professional	69	**0.46 **(0.36;0.58)	107	0.91 (0.75;1.10)
Military personnel	30	**0.42 **(0.30;0.61)	52	**0.53 **(0.40;0.69)

^1 ^Bold type, 95% CI does not include 1.00

^2 ^ICD codes: ICD-7: 240–289, ICD-8: 240–279, ICD-9: 240–279, ICD-10: E00-E90

^3 ^Adjusted for residential area, time period and fertility history

^4 ^Adjusted for residential area and time period

HR Hazard Ratio, CI Confidence Interval, NA not applicable

For both overall and cause-specific mortalities, the mortality of women showed a statistically significant association with socio-economic status. Compared with blue-collar workers, the HR for overall mortality was significantly elevated among farmhands (HR = 1.07, 95% CI 1.05–1.08) and it was particularly decreased in employers (HR = 0.78, 95% CI 0.76–0.79) and professionals (HR = 0.83, 95% CI 0.80–0.85). For men, service workers showed a significantly increased overall mortality (HR = 1.04, 95% CI 1.02–1.06) compared to the reference group blue-collar workers, whereas farmhands (HR = 0.94, 95% CI 0.93–0.95), farmers (HR = 0.85, 95% CI 0.85–0.86), white-collar workers (HR = 0.87, 95% CI 0.86–0.87), employers (HR = 0.85, 95% CI 0.83–0.86), professionals (HR = 0.93, 95% CI 0.91–0.95) and military personnel (HR = 0.83, 95% CI 0.81–0.85) showed a significantly decreased mortality (Table 1).

For overall cardiovascular disease mortality in women, farmhands were at a significantly increased risk (HR = 1.10, 95% CI 1.08–1.12) and employers showed the lowest risk (HR = 0.68, 95% CI 0.66–0.70). For men, farmhands, farmers, white-collar workers, employers, professionals and military personnel showed significantly decreased overall cardiovascular disease mortality. A similar pattern among women was observed for ischaemic heart disease. In men, compared to blue-collar workers, service workers, farmhands, farmers, white-collar workers, employers, professionals and military personnel showed significantly decreased ischaemic heart disease mortality. For cerebrovascular disease in women, farmhand showed the highest mortality compared to the lowest mortality among employers. In men, farmers, white-collar workers, employers, professionals and military personnel showed significantly decreased mortality. A socio-economic gradient was also noticeable for other forms of heart disease in women and in men (Table 2).

For overall cancer mortality in women, farmers, white-collar workers, employers, entrepreneurs and professionals showed significantly decreased mortality. For overall cancer mortality, we observed the highest mortality in men among service workers and the lowest mortality among farmers. A socio-economic gradient was also noticeable for lung cancer and stomach cancer in men and women. In women, only farmers were at a significantly decreased mortality due to colorectal cancer. Similarly in men, farmers showed the only decreased mortality and entrepreneurs the only increased mortality. For pancreatic cancer, no significant association between socio-economic status and mortality among women was observed. In men, farmers showed the only decreased mortality and entrepreneurs the only increased mortality due to pancreatic cancer. In contrast to other causes of death, increased mortality due to breast cancer in women was observed among white-collar workers and decreased mortality among farmhands. Increased mortality due to prostate cancer in men was observed among farmers and service workers (Table 3 and Table 4).

For overall respiratory disease, the highest mortality in women was observed among farmhand and the lowest in white-collar workers and employers. A socio-economic gradient was also observed among men. COPD showed large differences in mortality among female socio-economic groups (a 21% increase for service workers versus a 49% decrease in farmers) and male socio-economic groups (a 22% increase for service workers versus a 42% decrease in farmers). For influenza and pneumonia in women, farmhands showed the highest mortality and employers showed the lowest mortality. In men, farmhands, farmers, white-collar workers, employers, entrepreneurs, professionals and military personnel showed significantly decreased mortality (Table 5).

The socio-economic status was also significantly associated with mortality related to overall endocrine, nutritional and metabolic diseases in women and men. For example in women, employers showed a 58% decrease in mortality and farmhands an increase in mortality of 21%, both compared to blue-collar workers. In men, service workers showed a 28% mortality increase compared to a 44% mortality decrease for military personnel. A similar pattern was observed for diabetes mellitus in women with a 67% decreased mortality for employers and a 20% increased mortality for farmhands. For men, service workers showed a 35% increase in mortality and military personnel showed a 47% decrease in mortality (Table 6).

Spearman correlation coefficients between HRs for women and men according to disease from Tables 1 to Table 6 are shown in Table 7. Only socio-economic groups with significant HRs in at least one gender were included. Furthermore, the reference category of blue-collar workers was not included in the analysis. Significant correlation coefficients were observed for overall cardiovascular disease, ischaemic heart disease, cerebrosvascular disease and other forms of heart disease. Further, only lung cancer, overall endocrine, nutritional and metabolic diseases showed a significant correlation between men and women (Table 7).

**Table 7:** Comparison between women and men: Correlation between HRs for women and men according to disease^1^

Disease	Spearman coefficient	p value
**Overall mortality**	0.60	0.115

**Cardiovascular disease**	**0.77**	0.041
Ischaemic heart disease	**0.76**	0.028
Cerebrovascular disease	**0.71**	0.048
Other forms of heart diseases	**0.92**	0.003
		
**Cancer**	0.57	0.136
Lung cancer	**0.96**	<0.001
Colorectal cancer	0.50	0.667
Stomach cancer	0.87	0.873
Pancreatic cancer	-0.50	0.667
		
**Respiratory disease**	0.34	0.405
COPD	0.55	0.160
Influenza and pneumonia	0.27	0.558
		
**Endocrine, nutritional and metabolic diseases**	**0.69**	0.05
Diabetes Mellitus	**0.76**	0.028

^1 ^Socio-economic groups with significant HRs in at least one gender are included

**Figure 1:**
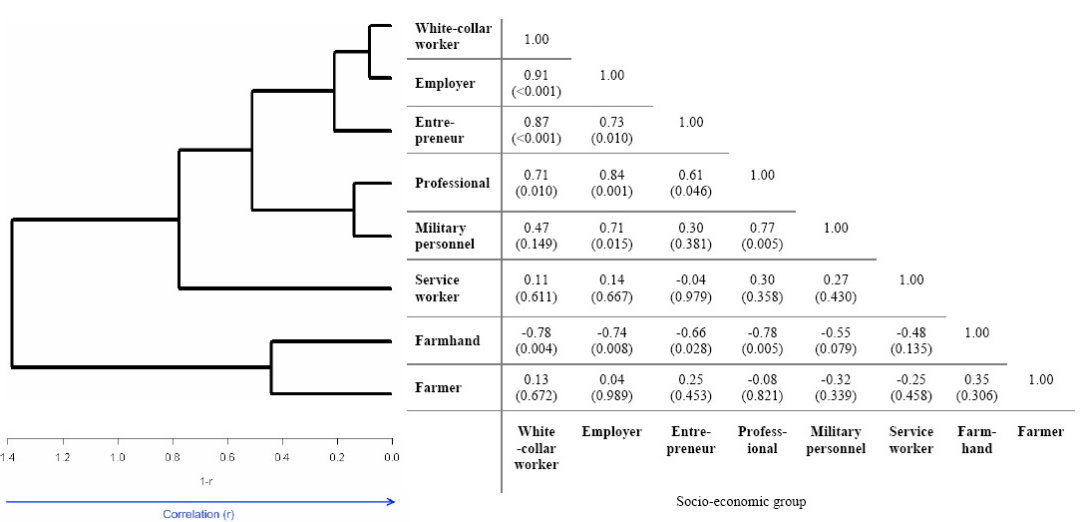
**Spearman correlation and clustering of HRs for women**. Spearman correlation analysis between HRs (r, p value) and hierarchical clustering dendogram with correlation-based distance (1-r) for women.

**Figure 2:**
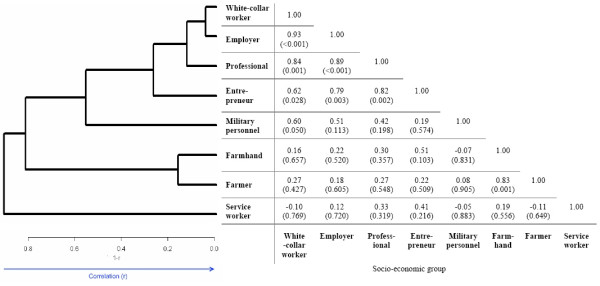
**Spearman correlation and clustering of HRs for men**. Spearman correlation analysis between HRs (r, p value) and hierarchical clustering dendogram with correlation-based distance (1-r) for men.

The results of the Spearman correlation analysis and the dendrograms showing average-linkage hierarchical clustering of female and male socio-economic groups are shown in Figure 1 and Figure 2. The distance metric used was 1-r, where r is the Spearman correlation coefficient between socio-economic groups.

## Discussion

This population-based study showed significant differences in mortality by socio-economic status for both men and women in the Swedish population. Socio-economic mortality differences in women were the most profound for lung cancer, COPD, overall endocrine, nutritional and metabolic disease, and diabetes mellitus, with the lowest HR for diabetes mellitus among female employers and the highest HR for lung cancer among female service workers. In men mortality differences were the most profound for lung cancer, COPD, overall endocrine, nutritional and metabolic disease, and diabetes mellitus, with the lowest HR for lung cancer among male farmers and the highest HR for diabetes mellitus among male service workers.

In our analysis we used overall and cause-specific mortality as the outcome. Some other studies have investigated socio-economic variation in survival following the diagnosis of a specific disease, such as cancer [17,21,32-39], ischaemic heart disease [40-42] or stroke [43,42]. These studies generally reported improved survival in many countries for individuals with higher socio-economic status, compared to individuals with lower socio-economic status. Several possible explanations for the observed survival differences among socio-economic groups have been proposed, such as earlier diagnosis in individuals with higher socio-economic status, consequently leading to a longer interval between diagnosis and death (lead time bias) [19,44,18,17], and differences in treatment among socio-economic groups [38,17]. On the other hand, some studies showed no or only a small relationship between socio-economic status and stage at diagnosis [45-47]. Additionally, adverse behavioural and lifestyle aspects have been reported to be more common among more deprived groups [48,49].

Table 8 provides a short summary of the causes of death discussed in the following section along with the socio-economic groups with the highest and lowest observed mortality risk and potential determinants. The socio-economic status was strongly related to overall cardiovascular disease and ischaemic heart disease, showing decreased mortality risk for both male and female farmers, white-collar workers, employers, entrepreneurs, professionals and military personnel. In contrast to female service workers and farmhands, male service workers and farmhands were at decreased mortality risk from overall cardiovascular disease and ischaemic heart disease. Socio-economic inequalities in mortality from overall cardiovascular disease and ischaemic heart disease were also reported by previous studies [1,4,3,41,7]. Our results might reflect different distributions of risk factors for overall cardiovascular disease and ischaemic heart disease, varying among female and male socio-economic groups.

**Table 8:** Relationship between causes of death and socio-economic groups: Summary of the relationship between causes of death, female and male socio-economic groups with highest and (↑) and lowest (↓) mortality and potential determinants. Please refer to the text for a more detailed discussion.

Cause of death	Socio-economic group with highest/lowest mortality risk	Potential determinates
**Cardiovascular disease**	female farmhand ↑ female employer ↓ male military personnel ↓^1^	Risk factors (high blood pressure, smoking, physical inactivity and obesity) varying among female and male socio-economic groups
**Lung cancer**	female service worker ↑ female farmer ↓ male farmer ↓^1^	Smoking
**Prostate cancer**	male service worker ↑^1^	Low PSA screening
**Breast cancer**	female white-collar worker ↑ female farmhand ↓	Access to screening, stage at diagnosis, reproductive history, age at first parturition, hormone replacement therapy
**Overall respiratory disease**	female farmhand ↑ female white-collar worker ↓ male service worker ↑ male military personnel ↓	Smoking, air pollution, allergens
**COPD**	female/male service worker ↑ female/male farmer ↓	Smoking, exposure to pollutants, allergies and asthma
**Overall endocrine nutritional and metabolic disease/diabetes mellitus**	female farmhand ↑ female employer ↓ male service worker ↑ male military personnel ↓	Overweight, smoking, physical inactivity

^1 ^With blue-collar worker as reference category no other group showed increased/decreased mortality risk

The association between lung cancer and socio-economic status differed for women and men. Female service workers were at an increased risk and female farmhands and farmers were at a decreased risk of lung cancer mortality. We equally observed a decreased risk among men except for service worker and military personnel. As smoking is a major risk factor for lung cancer [60], our observed differences might reflect changing patterns of smoking among socio-economic groups and among men and women in Sweden [12,6].

We observed increased mortality due to prostate cancer among male farmers and service workers, compared to male blue-collar workers. No further socio-economic group was at a significant risk of prostate cancer mortality. For prostate and breast cancer higher rates in incidence and mortality has been reported among more advantageous groups [13,49-53]. It was argued that greater take up of PSA screening and earlier detection of lesions among men with higher socio-economic status or education (lead time bias) could at least partly explain the inverse gradient observed in prostate cancer incidence [51,38]. In an earlier case-control study in Sweden, socio-economic status was not associated with risk of prostate cancer [54], and also other studies reported no clear association [55,56]. Many factors have been associated with prostate cancer that were not recorded in the national censuses, including obesity, physical exercise, diet, tobacco use, and alcohol use [57,58], which could partly explain our results.

Although delayed childbearing and nulliparity are known to be high risk factors for breast cancer [59], only a few studies have assessed the impact of fertility history on the inverse socio-economic gradient observed in breast cancer incidence and mortality [3,53,13,2]. After adjusting for fertility history we observed an increased mortality due to breast cancer among white-collar workers and a decreased mortality among service workers and farmhands. A previous Swedish study reported a significant inverse socio-economic gradient, still being significant after controlling for fertility history [3]. In a recent Danish study breast cancer incidence was higher in women with higher socio-economic status than in women with lower socio-economic status, which also persisted after adjusting for fertility history. However, there was a less pronounced gradient in breast cancer mortality and after controlling for fertility history none of the variations by socio-economic group remained significant [53]. On the other hand, Strand et al. observed that after adjusting for fertility history the educational gradient in breast cancer mortality among parous women disappeared. They concluded that fertility history or factors associated with it can fully explain the educational differences in breast cancer mortality among parous women in Norway. Further on they concluded that for nulliparous women, other factors than fertility history must explain the educational gradient observed [13].

Large differences in mortality between socio-economic groups were also found for overall respiratory disease and COPD among men and women. While female farmhands showed a slightly increased mortality due to overall respiratory disease, male farmhands showed a decreased mortality due to overall respiratory disease and COPD. A marked socio-economic gradient in overall respiratory disease and COPD, which was stronger in males and independent of smoking, was also previously reported by two Danish studies [10,20]. The gradient may be partly explained by differences in environmental and occupational exposure of women and men and among different socio-economic groups [10,61]. Lung cancer and COPD share the same common risk factors, which include smoking, genetic predisposition, and environmental and occupational exposures [62,63]. Among women we observed a significant correlation between mortality risk due to COPD and lung cancer (Spearman correlation coefficient = 0.79, p = 0.021), but not among men (Spearman correlation coefficient = 0.50, p = 0.207). This suggests a similar influence of the socio-economic status on COPD and lung cancer mortality among women, but not among men.

A marked socio-economic gradient was also observed for overall endocrine, nutritional and metabolic diseases, and diabetes mortality among women and men. Differences in HRs between socio-economic groups were more pronounced for women than for men. Large socio-economic inequalities in the prevalence and mortality of diabetes were also previously reported [64-67]. The explanation for this socio-economic gradient is unclear, but probably reflects increased exposure to lifestyle and environmental risk factors of diabetes for people with lower socio-economic status [64,66]. As type 2 diabetes and cardiovascular disease share common risk factors such as body mass index, physical activity, alcohol intake, and cigarette smoking [64,68,66], a significant correlation between mortality risk due to diabetes mellitus and cardiovascular disease was observed in women (Spearman correlation coefficient = 1.00, p < 0.001) and men (Spearman correlation coefficient = 0.95, p < 0.001), suggesting that socio-economic status is associated with a certain lifestyle in women and men.

The correlation analysis encompassed the most common diseases in the Swedish Family-Cancer Database, suggesting similar influence of the socio-economic status on cause-specific mortality, such as cardiovascular diseases, lung cancer, endocrine, endocrine, nutritional and metabolic diseases. The cluster analysis grouped female and male socio-economic groups with similar mortality risks. For both women and men farmer and farmhand as well as white-collar worker and employer were considered the most similar in mortality. These results might help to identify further differences in cause-specific mortalities between female and male socio-economic groups.

The present population-based study had several methodological strengths. First, it encompassed over 2 million men and women, and covered a follow-up period of over 40 years. Second, reporting bias was eliminated as all variables were based on register data from the Swedish Family-Cancer Database. As census forms are individually filled out, however, they may contain small inaccuracies. Further, registration of causes of death was highly complete and was obtained from death certificates from the Swedish Causes of Death Register. However, some limitations must be considered when interpreting the present results. Although the socio-economic group blue-collar worker was used as reference category in each model, the job/function description might differ by sex. This issue should be considered when comparing findings for women and men. Most importantly, the lack of potential risk factors not included in the national registries, such as use of hormonal contraceptives, age at menarche, alcohol consumption, body mass index, tobacco use, physical activity etc., preclude the estimation of their effect on overall and cause-specific mortality. Further, it is important to point out here that the structure of the Swedish Family-Cancer Database (Swedes born after 1931 with their biological parents), together with the restriction of the analyses to individuals aged 30–60 years in 1960, resulted in a study population were all individuals were parents and probably excluded adults with severe health problems. Furthermore, we were not able to investigate the socio-economic influence for nulliparous women. In order to account for changes in treatment over time, we adjusted our models for time period. Comparisons with other results on mortality must also take into account that our study population only consisted of individuals with a documented socio-economic status and probably did not include unemployed individuals.

The examination of the Database showed that about 70% of the individuals belonged to the same socio-economic group in the 1960 and the 1970 censuses. To evaluate the impact of the change in socio-economic position on the calculated HRs, we replicated the analyses for the first 10 years of follow-up (1960 to 1970). As expected, the relationship between socio-economic status and mortality was stronger during the first 10 years of follow-up for most causes of death. For example, the HR for overall respiratory disease in female farmhand was HR = 1.44, 95% CI 1.07–1.95 in the first 10 years, compared to HR = 1.09 (95% CI 1.03–1.16) for the whole period of follow-up. The HR for any cause of death in male service worker was 1.12 (95% CI 1.05–1.19) for the first 10 years, compared to HR = 1.04 (95% CI 1.02–1.06) for the whole period. The correlation between the HRs restricted to the first ten years and the estimates for the overall period were 0.7 (p < 0.001) for women and 0.4 (p = 0.02) for men. Another limitation due to space was the consideration of any age at death. It is important to mention here that the HRs may vary with age due to changes in incidence, age-related prognosis and treatment. For example, in contrast to mortality due to breast cancer at any age, no significant relationship was observed between socio-economic status and mortality before age 50 years (results not shown here). A recent article [16] shows that socio-economic inequalities in breast cancer mortality disappeared between 1968 and 1996 in France. The investigation of the temporal trends in socio-economic differences of mortality warrants further investigation.

## Conclusion

The present study shows that in Sweden, a country with in principal universal access to health care, socio-economic status is significantly associated with overall and cause-specific mortality risk and social inequalities exist. Using the Swedish Family-Cancer Database we were able to investigate more specific causes of death than have been typically reported. Our results might reflect different behavioral and lifestyle aspects and different exposure to occupational and environmental factors among socio-economic groups with elevated overall and cause-specific mortality. Comparison of overall and cause-specific mortality among female and male socio-economic groups may provide helpful insights into the underlying causes of socio-economic inequalities in mortality. In addition, further research is needed to confirm our results and to identify specific factors related to increased mortality in specific socio-economic groups. These factors will help to prevent higher mortality among more deprived socio-economic groups in Sweden.

## Abbreviations

HR: Hazard Ratio; CI: Confidence Interval; COPD: Chronic Obstructive Pulmonary Disease; ICD: International Classification of Disease.

## Competing interests

The authors declare that they have no competing interests.

## Authors' contributions

MW conducted the analysis and wrote the manuscript, JLB supervised the analysis and commented on the manuscript, KS commented on the manuscript, JS provided the data and commented on the manuscript, KH designed the study and commented on the analysis and the manuscript. All authors have accepted the final manuscript.

## Pre-publication history

The pre-publication history for this paper can be accessed here:


